# Evaluation of an educational intervention (edworkcases) involving clinical cases and Nursing students: a cross-sectional observational study^1^

**DOI:** 10.1590/1518-8345.6190.3723

**Published:** 2023-01-06

**Authors:** María Isabel Guzmán-Almagro, Cristina Oter-Quintana, Carmen Clara Martín-Salinas, María Luisa Cid-Galán, Elena Carrillo-Camacho, María Victoria Navarta-Sánchez, Oscar Castedo-Martínez, María Teresa Alcolea-Cosín, Luciana Mara Monti Fonseca, Ana Isabel Parro-Moreno

**Affiliations:** 1 Autonomous University of Madrid, Nursing Department, Madrid, Madrid, Spain.; 2 La Paz University Hospital, Madrid, Madrid, Spain.; 3 Puerta de Hierro-Segovia de Arana Health Research Institute, Nursing and Health Care Research Group, Majadahonda, Madrid, Spain.; 4 Rey Juan Carlos University, Faculty of Health Sciences, Alcorcón, Madrid, Spain.; 5 Puerta de Hierro-Majadahonda University Hospital, Majadahonda, Madrid, Spain.; 6 Universidade de São Paulo, Escola de Enfermagem de Ribeirão Preto, PAHO/WHO Collaborating Centre for Nursing Research Development, Ribeirão Preto, SP, Brazil.

**Keywords:** Clinical Competence, Decision-Making, Problem-Based Learning, Nursing Students, Nursing Diagnosis, Standardized Nursing Terminology, Competência Clínica, Tomada de Decisões, Aprendizagem Baseada em Problemas, Estudantes de Enfermagem, Diagnóstico de Enfermagem, Terminologia Padronizada em Enfermagem, Competencia Clínica, Toma de Decisiones, Aprendizaje Basado en Problemas, Estudiantes de Enfermería, Diagnóstico de Enfermería, Terminología Normalizada de Enfermería

## Abstract

**Objective:**

to evaluate the impact of the (edworkcases) educational intervention on students’ evaluation outcomes in their clinical practices, their attitudes towards Nursing diagnoses, and their satisfaction.

**Method:**

this study used a cross-sectional observational design. The participants were 69 third-year Nursing students from a public university in Madrid, Spain. The data analysed in the study were the grades obtained by students for their clinical practices, as well as pre-post intervention scores on the Positions on Nursing Diagnosis Scale and a satisfaction survey. A means comparison by participation in the project (yes/no) was carried out using Student’s *t*-test. A means comparison by professor was conducted using Kruskal-Wallis tests.

**Results:**

participation rate: 72.4%; 92% of the participants were women; median age = 21 years old. Statistically significant differences were found between participants and non-participants in terms of mean score in the Overall Evaluation and in the Case Study Evaluation, with higher scores found among the group of participants. The mean score for attitudes towards Nursing diagnoses was 99.9 (SD=2.8) before the intervention and 111.1 (SD=2.9) after the intervention [95% CI: 3.3-19.2].

**Conclusion:**

the use of (edworkcases) as part of the practical training was considered satisfactory, enabling theory and practice to be combined and improving students’ attitudes towards Nursing diagnoses.

## Resumo

**Objetivo:**

avaliar o impacto da intervenção educacional (*edworkcases*) nos resultados da avaliação dos alunos em suas práticas clínicas, suas atitudes em relação aos diagnósticos de Enfermagem e sua satisfação.

**Método:**

estudo observacional transversal, realizado com 69 estudantes do terceiro ano de Enfermagem de uma universidade pública de Madri, Espanha. Os dados analisados foram obtidos das notas dos alunos em suas práticas clínicas, bem como, os escores pré- e pós-intervenção na Escala de Posições frente ao Diagnóstico de Enfermagem e uma pesquisa de satisfação. A comparação de médias por participação no projeto (sim/não) foi realizada por meio do teste *t* de Student. A comparação de médias por professor foi realizada por meio de testes de Kruskal-Wallis.

**Resultados:**

taxa de participação: 72,4%; 92% dos participantes eram mulheres; mediana de idade de 21 anos. Foram encontradas diferenças estatisticamente significativas entre participantes e não participantes em termos de pontuação média na Avaliação Geral e na Avaliação do Estudo de Caso, com maiores pontuações encontradas entre o grupo de participantes. A pontuação média das atitudes em relação aos diagnósticos de Enfermagem foi de 99,9 (DP=2,8) antes da intervenção e 111,1 (DP=2,9) após a intervenção [IC 95%: 3,3-19,2].

**Conclusão:**

a utilização de *edworkcases* como parte do treinamento prático foi considerada satisfatória, permitindo a articulação teoria e prática e melhorando as atitudes dos alunos em relação aos diagnósticos de Enfermagem.

## Resumen

**Objetivo:**

evaluar el efecto de la intervención educativa (*edworkcases*) sobre los resultados de la evaluación de los estudiantes en sus prácticas clínicas, sus actitudes hacia los diagnósticos de Enfermería y su nivel de satisfacción.

**Método:**

en este estudio se empleó un diseño transversal y observacional. Los participantes fueron 68 estudiantes de tercer año de la carrera de Enfermería de una universidad pública de Madrid, España. Los datos que se analizaron en el estudio fueron las calificaciones obtenidas por los estudiantes en sus prácticas clínicas, al igual que las puntuaciones antes y después de la intervención en la Escala de Posicionamiento ante el Diagnóstico de Enfermería y en una encuesta de satisfacción. Se utilizó la prueba *t* de Student para realizar una comparación de valores medios por participación en el proyecto (sí/no). La comparación de valores medios por profesor se llevó a cabo por medio de pruebas Kruskal-Wallis.

**Resultados:**

índice de participación: 72,4%; el 92% de los participantes eran mujeres, con una mediana de edad de 21 años. Se encontraron diferencias estadísticamente significativas entre participantes y no participantes en cuanto a la puntuación media en la Evaluación General y en la Evaluación de Estudios de Caso, con puntuaciones más elevadas en el grupo de participantes. Las puntuaciones medias correspondientes a las actitudes con respecto a los diagnósticos de Enfermería fueron 99,9 (SD=2,8) y 111.1 (SD=2.9) antes y después de la intervención, respectivamente [IC 95%: 3,3-19,2].

**Conclusión:**

se consideró satisfactorio utilizar *edworkcases* como parte te la capacitación práctica, lo que permitió combinar teoría y práctica y mejorar las actitudes de los estudiantes con respecto a los diagnósticos de Enfermería.

**Highlights**

(1) edworkcases is an intervention for the resolution and oral presentation of a case.

(2) The students report that it helps them in problem-solving and critical thinking skills.

(3) edworkcases appears to improve Nursing students’ attitudes towards Nursing diagnoses.

(4) The intervention requires professors trained in the use of the Nursing ProNursing Process.

(5) edworkcases is part of practical training and allows theory and practice to be combined.

## Introduction

Clinical practices are a key component of undergraduate training in Nursing. In accordance with the guidelines established by the European Higher Education Area (EHEA), the students must complete almost 40% of their overall training in real clinical settings in order to qualify for the BSc Nursing Degree. The last three academic years of the course consist of 6 practical modules in which the students engage in clinical training in simulation rooms and inpatient units or primary care facilities, mentored by clinical nurses and lecturers from the Nursing Department.

The fundamental objective of clinical practices is to prepare the students for the professional practice through acquisition of professional skills. Experiential learning in clinical settings may be described as a process that is: 1. self-directed, as the students select the area that most interests them based on their learning motivations and objectives; 2. collaborative, drawing on interaction with professionals, mentors and peers; 3. conditioned by the context in which it is carried out; and 4. meaningful, based on multiple experiences that generate meaning in the knowledge construction process^1^. In order to achieve some of the practice-related learning objectives, it is necessary to use methodologies that can leverage the maximum learning value from these clinical settings, such as case studies. There is a considerable body of research on the positive results of using case studies in simulation settings, such as in Objective Structured Clinical Examinations (OSCEs)^2^ ^-^ ^3^. 

Case studies have traditionally been used to train Nursing students in using the Nursing ProNursing Process and standardised Nursing languages, and they are considered an efficient, effective and practical tool for teaching diagnostic reasoning^4^ ^-^ ^5^. Case studies require students to apply their knowledge, skills and attitudes, not only to collect data, but also to transform these data into information^4^ and formulate diagnostic hypotheses^6^ ^-^ ^7^ as a preliminary step to planning patient outcomes and individualised Nursing interventions. 

Taking into consideration the potential association between the students’ attitudes towards Nursing diagnoses and their practical application reported in previous studies^8^, case studies may have great potential when it comes to familiarizing the students with Nursing diagnoses, and may also improve their attitudes towards them, encouraging their use in the subsequent clinical practice.

For these reasons, in the Nursing Department of Autonomous University of Madrid, in order to improve clinical reasoning skills and the use of standardized languages during the clinical practices, the students are asked to prepare a clinical case consisting in designing a care plan for hospitalised individuals in their clinical practice unit. The students must develop the different phases of the Nursing ProNursing Process: assessment, diagnosis, planning, implementation and evaluation of their care plan^9^.

While preparing their case study, students and university lecturers hold two scheduled meetings to address any questions that may arise during the process. In the end, their work is graded by the module convenor in charge. That grade is part of the overall evaluation of the module, which also includes other components such as being assessed by the clinical nurse responsible for mentoring them at the unit, as well as a self-evaluation component. This results in a comprehensive and integrated assessment of their performance from a variety of perspectives.

However, experience from previous years suggests that students with little clinical experience face major difficulties, especially in clinical reasoning and care planning, which can exert a negative impact on their evaluation and attitudes towards using the Nursing ProNursing Process in their future career. This reflection led to the consideration that the complexity of preparing case studies requires interaction of interpersonal, technical and intellectual processes which, in turn, demand continuous and structured support and supervision by the faculty in order to foster significant learning, diagnostic skills and positive attitudes towards case resolution among the students.

To this end, a group of professors from the Nursing Department implemented an **Ed**ucational intervention of **Work**shop and debate on clinical **Cases** (edworkcases) aiming to continuously monitor the development process of the case studies, creating small groups as spaces for reflection aimed at promoting the development of clinical reasoning and effective case resolution. This small group mentoring approach aimed at fostering an atmosphere of trust, where questions, opinions and disagreements could be voiced freely among peers, as this was believed to motivate the students and enhance their reflective, dialogical and communicative skills^10^. In a study, serious difficulties were observed among Nursing students with regard to communication skills, and they strongly recommended using teaching strategies that could enhance the acquisition of communication skills, as they are an essential component of high-quality care^11^.

The intervention included a final oral presentation of the case study to the other group members. During the presentation, the students had to give an account of the clinical reasoning and decision-making processes they followed to solve their case study, while answering the questions posed by the teaching staff and their peers.

The main objective of the current study was to evaluate the impact of the (edworkcases) educational intervention on the students’ evaluation outcomes in their clinical practices and, as specific objectives, to assess their attitudes towards Nursing diagnoses and their satisfaction with this educational intervention.

## Method

### Study type

A cross-sectional observational study was carried out at three hospitals in the Spanish Public Health System where Nursing students conduct their clinical practices.

### Population

The sample comprised the entire student population attending the third year of the Nursing Course at a public university in Spain (n=69), who were completing seven-week clinical practices at medical-surgical units at three hospitals in the Spanish Public Health System.

Convenience sampling was used to ensure feasibility of the study. To this end, an in-person session was organised to inform all students conducting clinical practices at the three selected hospitals about the study. In the session, the study objectives and methodology were explained, as well as the voluntary nature of participation and confidentiality of the data obtained. The students who showed an interest in participating were given an information sheet about the study.

### Study variables

Sociodemographic variables: gender and age. Academic variables: mean scores obtained on the instruments used to evaluate the students, scores on the overall evaluation, mean scores obtained in the Positions on Nursing Diagnosis (PND) Scale, and scores in the satisfaction survey.

### Data collection

**Figure 1:**
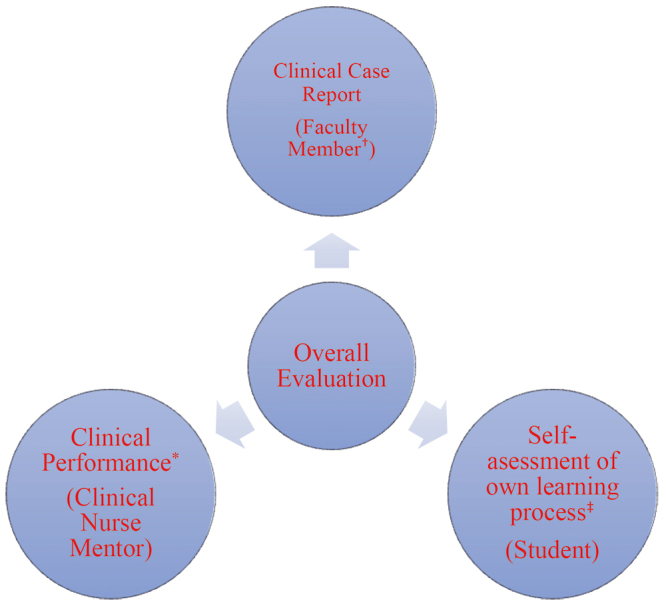
Components of the evaluation

^*^The Clinical Nurse Mentor (CNM) evaluates the students’ learning process and achievements in terms of their level of competence acquisition; ^†^The Faculty Member (FP) evaluates the students’ ability to apply theoretical knowledge to a case study; ^‡^Each student self-assesses their learning process and achievements (their own level of competence acquisition)

Data collection was carried out between January and May 2020. In order to obtain the results, the module’s evaluation instruments were used. This is an evaluation that seeks to be comprehensive and to include a variety of perspectives by way of having different components:

- Evaluation of the students’ learning during the clinical practice by their clinical mentor. This is a competence-based assessment document, structured in 7 dimensions, one of which concerns competences for applying the Nursing ProNursing Process.
- Self-evaluation by the students of their learning during the clinical practice. This document also includes a dimension regarding self-assessment in implementing the Nursing Process.
- Evaluation of the students’ academic work: a clinical case study. The research team designed an ad-hoc rubric for its evaluation (Figure 1).

The following scales were also taken into account for data analysis:

- Survey of student satisfaction with the case study workshop/debate methodology. The research team designed an ad-hoc survey for this study.
- The Positions on Nursing Diagnosis (PND) Scale. This scale allows students’ attitudes towards Nursing diagnoses to be measured and has been validated for use in the Spanish context with a Cronbach’s α of 0.96^12^ ^-^ ^13^. PND uses the semantic differential method^13^. It comprises 20 items, each of which contains two opposing adjectives representing the opposite extremes of a possible attribute of Nursing diagnoses. Positive and negative adjectives are situated at either end of the scale at random to avoid acquiescence. In each item, the attributes for both extremes are joined by a line divided into 7 equidistant points. The scores range from 1 to 7, with 1 representing the least favourable attitude and 7 the most favourable attitude towards Nursing diagnoses. The total score for the scale ranges from 20 to 140 points. Higher scores represent more positive attitudes towards Nursing diagnoses. The scale is a self-report measure.

The research team was made up of professors with experience in mentoring students on clinical practices and Nursing languages. The research team members designed the formal aspects of the intervention: number of sessions, days, and session content; composition of the student groups; consultation with the University’s Research Ethics Committee; and selection of the data collection instruments. They also presented the project to the other professors in the department and encouraged them to participate.

The new teaching intervention consisted of three scheduled small group meetings with approximately 8-9 students each, chaired by a lecturer from the research team, who worked full-time or part-time at the university, and who guided and moderated the group in order to promote a climate of trust for the oral presentation of the cases, addressing doubts and with discussion among peers. In addition, at the end of the trainee rotation programme, each student had to present their clinical case in a public hearing for professors, clinical mentors and other students.

**Figure 2:**
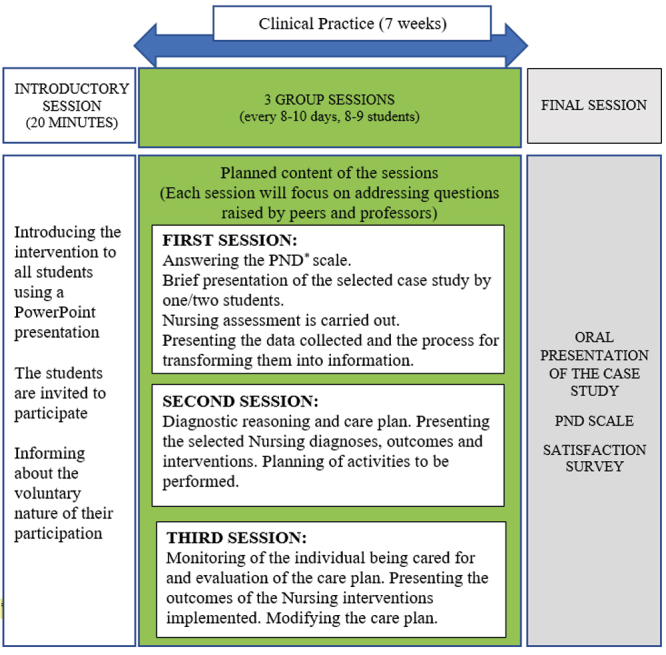
Educational intervention

^*^PND = Positions on Nursing Diagnosis Scale

The data collection strategy was applied during all three phases of the project: an *Introductory session* for students, where the project was presented and their participation was encouraged, the *Clinical practice period*, and the *Final session*. Further details of the intervention are shown in Figure 2.

### Data treatment and analysis

A descriptive analysis was carried out, and the means and standard deviations were calculated for the overall scores, the case study scores, the scores on the instruments used by clinical mentors to evaluate students, the scores on PND, and the scores on the satisfaction survey.

Student’s *t*-tests for independent samples were used to compare the mean scores for the evaluation items by participation in the study (yes/no). The Kruskal-Wallis test was used to compare mean scores for the clinical case by professor. Student’s *t*-tests for paired samples were used to compare mean scores on the diagnostic positioning scale before and after the intervention.

A 95% confidence interval was calculated and a significance level of 0.05 was used. The STATA 12 statistical software package was used for the analysis.

### Ethical aspects

The project was approved by the Call for Teaching Innovation at Autonomous University of Madrid (2019/2020 academic year). It was also approved by the Clinical practices Committee at the faculty, and the University Ethics Committee gave its consent for the study to go ahead. The students were informed about the objective and implications of the new teaching methodology and of the voluntary nature of their participation, both verbally and in writing. All students who decided to participate signed an informed consent form. For data collection, an alphanumerical code was assigned to each student to ensure confidentiality. The project adhered to the principles established by the Declaration of Helsinki (1964) and by Law No. 15/1999 on Personal Data Protection. 

## Results

Fifty out of 69 enrolled students participated in the educational intervention, representing a 72.4% participation rate. Three students were rejected for failing to complete the PND scale. Ninety-two percent of the sample were women, with a median age of 21 years old (minimum: 19; maximum: 57). The participation rate among the faculty was 75%.

The mean score in the Overall Evaluation for the module among the participants was 9.03 (SD=0.4) compared to 8.7 (SD=0.5) among the non-participants. Statistically significant differences were found between both groups (*p*<0.01). With regard to the Case Study Evaluation (CSE instrument), the mean score among the participants was 8.9 (SD=0.7) compared to 8.2 (SD=1) among the non-participants. Statistically significant differences were found between both groups (*p*<0.01). Statistically significant differences were also found in the case study scores depending on the teacher scoring them (Table 1).

**Table 1:** Mean scores for the evaluation items by participation and comparison of scores by mentor (n=69). Madrid, Spain, 2020

	Mean	SD^*^	Participants (yes/no)	Mean	SD^*^	Means difference	[95% CI^†^)]*Lower limit, upper limit*		*p*-value^§^
Overall evaluation	8.9	0.4	Yes (*n*=50)	9.03	0.4	-0.3	-0.5	-0.08	0.007
			No (*n*=19)	8.7	0.5				
Case study evaluation	8.7	0.9	Yes (*n*=50) No (*n*=19)	8.9 8.2	0.7 1	-0.6	-1.1	-0.2	0.009
Self-evaluation	9	0.6	Yes (*n*=50) No (*n*=19)	9.1 8.7	0.6 0.7	-0.3	-0.6	-0.02	0.05
**Case study evaluation by mentor**	**Professors**		**Participants**		**Median**		**P25 ^‡^**	**P75 ^‡^**	***p*-value^¶^**
	Professor 1		Yes		9		8.6	9.07	0.001
	Professor 2		Yes		8.6		7.5	9.07	
	Professor 3		Yes		9.3		9	9.3	
	Professor 4		Yes		8.3		7.7	8.6	
	Professor 5		Yes		9.1		8,8	9.3	
	Professor 6		Yes		8.8		7.5	8.9	
	Professor 7		No		9.1		8.8	9.4	
	Professor 8		No		9.5		9	9.8	

*SD = Standard Deviation; ^†^CI = Confidence Interval; ^‡^P = Percentile; ^§^Student’s *t*-test; ^¶^Kruskal-Wallis test

The mean score for the attitudes towards Nursing diagnoses among the students was 99.9 (SD=2.8) before the intervention and 111.1 (SD=2.9) after the intervention, with statistically significant differences identified between both scores (*p*<0.01). Table 2 shows the differences in the survey scores pre- and post-intervention, with higher scores (more positive attitudes) identified in 17 of the 20 items of the survey. Particularly pronounced differences in scores were observed for the following items: “ambiguous-clear”, with a difference of 0.9 points (*p*=0.01); “easy-difficult”: 0.9 (*p*=0.03); and “creative-routine”: 1.2 (*p*=0.00) (Table 2).

**Table 2:** Means comparison for items, overall scores, and by category of professor according to the PND scale pre- and post-intervention (n=47). Madrid, Spain, 2020

Items	Means (pre)	Means (post)	Differences	*p-*value^†^	[95% CI*, Lower and upper limit]	
1.Ambiguous-Clear	4.7	5.5	0.9	0.01	0.2	1.5
2.Meaningless-Meaningful	5.6	6.2	0.7	0.04	0.2	1
3.Pleasant-Unpleasant	4.7	5.2	0.5	0.07	-0.04	1
4.Strong-Weak	4.3	4.9	0.6	0.04	0.02	1.1
5.Valuable-Worthless	5.4	5.4	0	0.9	-0.7	0.7
6.Negative-Positive	5.9	5.9	0	0.8	-0.4	0.5
7.Stupid-Intelligent	5.7	6.2	0.4	0.02	0.05	0.8
8.Comfortable-Uncomfortable	4.5	5.2	0.8	0.01	0.2	1.4
9.Easy-Difficult	3.9	4.9	0.9	0.03	0.3	1.5
10.Unrealistic-Realistic	4.3	5.1	0.8	0.02	0.2	1.5
11.Helpful-Hindering	5.2	5.5	0.3	0.25	-0.2	0.8
12.Invalid-Valid	5.3	6	0.7	0.00	0.2	1.3
13.Meaningful-Meaningless	5.5	5.6	0.1	0.8	-0.6	0.8
14.Relevant-Irrelevant	5.6	5.7	0.1	0.7	-0.5	0.7
15.Unrewarding-Rewarding	4.9	5.6	0.7	0.00	0.2	1.2
16.Appropriate-Inappropriate	5.5	5.4	-0.1	0.5	-0.8	0.4
17.Acceptable-Unacceptable	5.4	5.7	0.2	0.4	-0.4	0.8
18.Bad-Good	5.8	6	0.1	0.5	-0.2	0.5
19.Creative-Routine	3.9	5	1.2	0.00	0.5	1.9
20.Unimportant-Important	5.8	6.1	0.3	0.18	-0.1	0.7
Overall score	99.9	111.1	11.2	0.00	3.3	19.2
Overall score for part-time professors	101.2	99.4	5.8	0.7	-14.3	10.6
Overall score for full-time professors	101.6	118.8	17.2	0.00	7.8	26.7

^*^CI = Confidence interval; ^†^Student’s *t*-test

Student satisfaction with the new teaching methodology obtained high scores. With regard to the educational intervention’s contribution to completion of the case study and skills acquisition, the items that scored above 4 on a scale from 0 to 5 (0=Completely disagree and 5=Completely agree) were the following: *It allowed me to apply theory to practice*, with a score of 4 (SD=0.97); *Autonomous learning*, with 4.2 (SD=0.97); *Information management*, with 4 (SD=0.95); *Critical thinking*, with 4.3 (SD=0.98); *Problem-solving skills*, with 4 (SD=1.04) (Table 3).

**Table 3:** Mean scores on the student satisfaction survey (n=35). Madrid, Spain, 2020

Items	Mean^*^	Standard deviation
**Part 1. Evaluate the methodology used to facilitate completion of the case study**		
It increased my motivation to complete the case study.	3.74	1.04
It allowed me to apply theory to practice.	4.00	0.97
It met the expectations I had when I received information about the project.	3.51	1.29
It was useful to me when carrying out this task.	3.89	1.02
It allowed me to improve my ability to use Nursing methodology and standardised Nursing languages during the clinical practice.	3.63	1.21
**Part 2. Evaluate the methodology used in relation to skills acquisition**		
Teamwork	3.17	1.12
Communication	3.94	0.97
Autonomous learning	4.23	0.97
Information management	4.03	0.95
Critical thinking	4.26	0.98
Clinical decision-making	4.03	1.04

^*^Range = 0-5. 0 = Lowest score and 5 = Highest score

## Discussion

According to the results obtained, using case studies as a teaching methodology during practical training appears to be a useful tool in Nursing students’ education. Learning based on case studies allows students to combine theory and practice, develop critical thinking skills, improve their problem-solving abilities, and apply an individualised approach to each case^14^ ^-^ ^16^.

In this study, the main differences between traditional methods and the new teaching methodology evaluated were the inclusion of an oral presentation of the case study and the application of collaborative learning. The use of an oral presentation describing a case study as an evaluation method has no precedent in the scientific literature, and some studies have aimed at analysing, assessing and confirming its real value^17^. It explains that oral presentations can improve both communication skills by reducing fear of public speaking and written expression through the process of planning and organising ideas when preparing the presentation^18^. Similarly, sharing a case study helps to cement the knowledge acquired^19^.

The results show significant differences in the scores obtained for the case study depending on the identity of the professor, despite the use of identical evaluation rubrics. Although one of the objectives of using a rubric is to minimise variability in the scores given by the professors for a particular task^20^, they do not always eliminate subjectivity, and differences owing to each teacher’s particular characteristics may persist^21^. Tools for evaluating the quality of the case studies produced, at least partially (such as Lunney’s Scale for Accuracy of Nursing Diagnoses)^12^, may have been usefully applied in this study to establish whether these differences in scores are primarily due to the quality of the case studies presented by the students and, therefore, to the academic heterogeneity of the groups assigned to each tutor, or to differences in application of the rubric.

The differences observed in the scores for the evaluation given by participating and non-participating mentors can be explained by the use of different rubrics in each case, despite attempts to ensure that the criteria for both evaluation tools coincided as much as possible.

The differences in the total scores obtained in the PND scale before and after the intervention indicate that the educational intervention improves the students’ attitudes towards Nursing diagnoses. This corroborates the findings of studies conducted with Nursing Professionals, whose scores increased after participating in activities aiming to improve their knowledge and clinical and diagnostic reasoning skills^22^. It also echoes the findings of other authors^13^ with regard to the value of the “use” of Nursing diagnoses (made possible by training interventions such as the one used in this study) in improving attitudes towards them. Although the students who completed the educational intervention with full-time teaching staff obtained higher scores on the scale than those working with part-time faculty, this finding can be coincidental and may be explained by the peculiar features of each group of mentees.

The mean overall scores obtained in the scale after the intervention were higher than those observed in other Nursing student populations^8^ ^,^ ^12^ and indicate a favourable attitude towards Nursing diagnoses. Establishing positive attitudes towards these diagnoses among Nursing students is central to improving adherence in the real clinical practice, although it is insufficient to ensure diagnostic competency^12^ ^,^ ^23^. Diagnosis “difficulty” is the lowest-scoring item on the scale, despite the noteworthy increase in the score for this item. This finding is in line with the results of studies carried out with nurses^24^ and students^8^ ^,^ ^12^. This may be explained by the complexity of the diagnostic reasoning process^25^ in which interpersonal, technical and intellectual processes interact^26^, and/or by the presence of limited training in Nursing diagnoses in Nursing education programmes^27^. The perception among the participants that Nursing diagnoses are “important” is shared by Nursing students^8^ and Nursing Professionals^24^. Alongside this attribute, “meaningful” and “intelligent” obtain the highest scores on the scale, suggesting that the participants would appear to perceive Nursing diagnoses as relevant to their professional practice.

It is important to note that student satisfaction with the new teaching methodology obtained high scores. According to the students, this learning experiment allowed them to apply theory to practice. This finding is of particular interest due to the traditional difficulty ensuring that the students transfer the theoretical knowledge from the theory modules studied to their clinical practices^28^. Similarly, the students observed that the learning experiment had allowed them to acquire skills such as problem-solving and critical thinking skills to a high level (scoring higher than 4 on a scale from 0-5). Development of critical thinking among Nursing students is considered essential to their performance in their future careers^29^. It encourages them to think and reflect in order to anticipate any complications that their patient may be likely to experience^30^ ^-^ ^31^. Therefore, these findings indicate that this new teaching methodology may be beneficial in improving Nursing students’ training in clinical practice modules.

However, it is relevant to note that the teamwork skill only obtained a moderate score in the satisfaction survey. Although this may be considered a weakness of the learning experiment, the potential of this method for collaborative learning compensates for this result.

In terms of costs, the project demanded a greater effort from the faculty than their normal teaching work. Requirements to ensure success of the intervention include that the teaching staff be motivated and trained in the use of the Nursing methodology and standardised Nursing languages.

The methodological limitations of the study include the use of convenience sampling. It was not possible to analyse reliability of the satisfaction survey due to the sample size, which may have resulted in lack of sensitivity of the instrument used. Attempts were made to control variability in the execution of the educational intervention deriving from the characteristics of the teaching staff participating in the study through a collaborative design of the teaching objectives and method, and the use of standard evaluation criteria by all participating professors to ensure homogeneity of the intervention in practice.

Personalised care requires the correct application of the care process and, more specifically, the correct identification of Nursing diagnoses in each patient. Further studies with larger sample sizes are needed to confirm these findings and to determine applicability of this educational intervention to Nursing students in other settings and countries. It would also be desirable to investigate the students’ learning performance regarding the identification of Nursing diagnoses in the clinical practice throughout university academic training, as well as to explore the degree of adherence to Nursing diagnoses among graduate students during their first years of their professional careers. It should also be emphasised that we believe that the results of this study can provide a deeper understanding of teaching methodologies in the Nursing science.

## Conclusion

The (edworkcases) intervention appears to improve Nursing students’ attitudes towards Nursing diagnoses. According to the students, the methodology encourages the development of soft skills essential to future Nursing ProNursing Professionals’ training, such as critical thinking and problem-solving. It also enables them to apply theory to practice. Practical implementation of the intervention requires preparatory work to ensure homogeneity in its execution by the teaching staff, as well as to develop evaluation tools that mitigate the evaluators’ subjectivity. In addition, student satisfaction with the new teaching methodology obtained high scores.
